# Identification of biological pathways and genes associated with neurogenic heterotopic ossification by text mining

**DOI:** 10.7717/peerj.8276

**Published:** 2020-01-03

**Authors:** Yichong Zhang, Yuanbo Zhan, Yuhui Kou, Xiaofeng Yin, Yanhua Wang, Dianying Zhang

**Affiliations:** 1 Department of Trauma and Orthopaedic Surgery, Peking University People’s Hospital, Beijing, China; 2 Department of Periodontology and Oral Mucosa, the Second Affiliated Hospital of Harbin Medical University, Harbin, Heilongjiang, China

**Keywords:** Heterotopic ossification, Spinal cord injury, Traumatic brain injury adipogenesis, Text mining

## Abstract

**Background:**

Neurogenic heterotopic ossification is a disorder of aberrant bone formation affecting one in five patients sustaining a spinal cord injury or traumatic brain injury (SCI-TBI-HO). However, the underlying mechanisms of SCI-TBI-HO have proven difficult to elucidate. The aim of the present study is to identify the most promising candidate genes and biological pathways for SCI-TBI-HO.

**Methods:**

In this study, we used text mining to generate potential explanations for SCI-TBI-HO. Moreover, we employed several additional datasets, including gene expression profile data, drug data and tissue-specific gene expression data, to explore promising genes that associated with SCI-TBI-HO.

**Results:**

We identified four SCI-TBI-HO-associated genes, including GDF15, LDLR, CCL2, and CLU. Finally, using enrichment analysis, we identified several pathways, including integrin signaling, insulin pathway, internalization of ErbB1, urokinase-type plasminogen activator and uPAR-mediated signaling, PDGFR-beta signaling pathway, EGF receptor (ErbB1) signaling pathway, and class I PI3K signaling events, which may be associated with SCI-TBI-HO.

**Conclusions:**

These results enhance our understanding of the molecular mechanisms of SCI-TBI-HO and offer new leads for researchers and innovative therapeutic strategies.

## Introduction

Heterotopic ossification (HO), also known as heterotopic bone formation, is the process of benign bone formation and growth outside of normal skeletal locations (Sullivan et al., 2013). Neurogenic heterotopic ossification (NHO) was first described at the end of the nineteenth century and was predominantly observed among soldiers who had experienced spinal cord trauma during combat in World War I (Shehab, Elgazzar & Collier, 2002). At present, three subtypes of NHO have been described: traumatic, neurologic, and genetic. The latter subtype derives from a rare hereditary disorder associated with NHO. The first two subtypes, by contrast, are more common and are acquired following various traumas, fractures, dislocations, elective orthopedic surgeries (e.g., hip surgery), electrocution and burn injuries, and neurological damage. Specifically, neurologic NHO refers primarily to the spinal cord and traumatic brain injury. In spinal cord-injured patients, the incidence of ectopic bone formation is between 10% and 25% (Sakellariou et al., 2012; Vanden Bossche & Vanderstraeten, 2005), with most formation occurring in the hip region. Ectopic bone formation occurs in 40–50% of patients with traumatic brain injury, with the hip (Sakellariou et al., 2012), shoulder, and elbow being common sites of formation. Clinically, NHO caused by traumatic brain injury always affects the joints of both upper and lower extremities, but NHO caused by spinal cord injury always occurs predominantly below the level of the lesion. Ossification is also generally seen anteriorly involving the iliopsoas and femoral neurovascular structures, laterally within gluteus minimus, and posteriorly extending from the ilium to the posterior femur encasing the sciatic nerve (Sullivan et al., 2013). The occurrence of NHO is problematic for patients with brain and spinal cord injuries, as it can cause pain, inflammation, and loss of range of motion, as the joint gradually becomes ankylosed, leading to greater functional disability (Genêt et al., 2011). Although NHO has long been reported, and the relevant literature is continuously growing, the etiology and mechanisms of neurologic NHO have not yet been recognized. There are currently no efficacious means of prevention and few treatment options for neurologic NHO. Once acquired NHO develops, surgical removal is the only effective treatment, and is usually followed by local radiation or nonsteroidal anti-inflammatory drugs (NSAIDs) to prevent recurrence (Sullivan et al., 2013; Cullen & Perera, 2009). Indomethacin is now the gold standard for pharmacological prevention of NHO (Sullivan et al., 2013). Another drug, rofecoxib, significantly decreases the rate of NHO and has been used as a primary prevention strategy for NHO, although it was taken off the market by the United States Food and Drug Administration in September 2004 (Sullivan et al., 2013). Moreover, the efficacy of bisphosphonates (specifically etidronate) as a primary prevention strategy for neurogenic NHO in patients with spinal cord and traumatic brain injury has been reported and reviewed (Sullivan et al., 2013; Sakellariou et al., 2012). The lack of preventative and treatment options may be attributed to a lack of accepted animal models (Kan & Kessler, 2011), and many researchers only investigate and report the epidemiological features (incidence, recurrence, and localization in patients) (Genêt et al., 2011), although still in the experimental stages Reznik et al. (2017a, 2017b) report upon the novel intervention of extracorporeal shock wave therapy for the management/treatment of NHO in TBI.

Over the course of time, the biomedical literature has rapidly expanded. When clinicians and researchers search the literature for relevant information (e.g., the genes or pathways associated with HNO), they are often faced with a common problem: there is such a large quantity of literature that it is difficult to collate all the relevant information in their field, and even more so outside it (Hristovski, Rindflesch & Peterlin, 2013). Fortunately, text mining technology can be used as an approach to gain a broad understanding of an entire dataset and the dynamics of the study issues. More importantly, text mining can pose a hypothetical question or find potential relationships (that may otherwise be ignored) between entities of interest. One relevant area of research is literature-based discovery (LBD); some researchers have successfully used this strategy to promote new discoveries for diseases (Hristovski, Rindflesch & Peterlin, 2013; Karic et al., 2011; Swanson, 1986; Karić & Karić, 2011; Zhan et al., 2017; Swanson, 1988). One of the most well-known examples of the successful use of LBD is the discovery that patients with Raynaud’s disease could benefit from the consumption of fish oils (Swanson, 1986).

**Figure 1:**
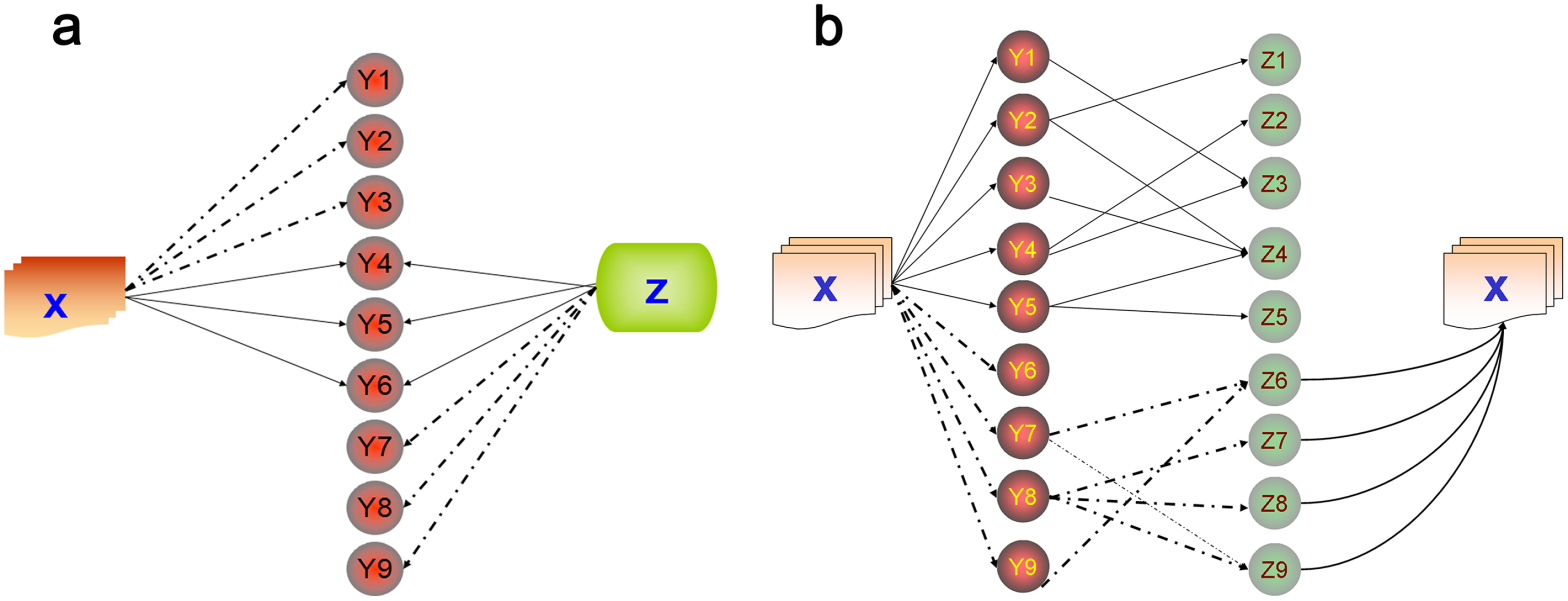
The two main approaches for performing literature-based discovery (LBD): closed discovery and open discovery. (A) In closed discovery, the search process starts simultaneously from terms X (e.g., Heterotopic ossification) and Z (e.g., Spinal Cord Injuries or Traumatic Brain Injuries), resulting in overlapping Ys. In this example, Y4, Y5, and Y6 may play key roles in the communication between spinal cord injury or traumatic brain injury and heterotopic ossification. The dashed arrows indicate unsuccessful “Gene or Gene Product” terms. (B) In open discovery, the search process starts from X (e.g., Heterotopic ossification), then the second concept (Y, e.g., Spinal Cord Injuries, Traumatic Brain Injuries) is searched. Next, the related “Z” concepts (e.g., “Gene or Gene Product”) are explored by defining the semantic type (e.g., as “Genes or Molecules.”) Finally, the relationship between Z and X is verified. If these “Z” terms (Z1, Z2, Z3, Z4 and Z5) have not been mentioned/tested in the literature, then they may be potential new genes for X, and necessitate further study. The dashed arrows indicate unsuccessful new discoveries, since Z6, Z7, Z8 and Z9 have already been associated with X.

Nearly all LBD systems are inspired by or derived from Swanson’s ABC co-occurrence model (Swanson, 1988; Henry & McInnes, 2017). The methods and evolution of LBD have been introduced and reviewed elsewhere (Henry & McInnes, 2017; Hristovski et al., 2005). Briefly, “A implies B” and “B implies C” relationships are generated by the explicit knowledge that already exists in the literature, then implicit knowledge is discovered by drawing a “therefore A implies C” conclusion (Henry & McInnes, 2017). There are two main approaches to LBD: open discovery and closed discovery (Fig. 1) (Hristovski et al., 2006). These two methodologies have different focuses. Open discovery requires a single term/concept (“A,” e.g., a disease), and then first-order terms that relate to the single term/concept are categorized (“B,” e.g., drug). Then, those first-order concepts are further categorized (“C,” e.g., gene). Thus, information flow can be linked from “A” (disease) to “B” (drug), then to “B” (drug) to “C” (gene). If these “C” (gene) terms have not been mentioned in the literature, then they are potentially related to “A” and “B,” but are untested. Closed discovery requires a start term/concept and a target term/concept, and then seeks intermediate links to generate potential explanations for the relationship between two terms/concepts (start and target) (Hristovski et al., 2006). Hristovski, Rindflesch & Peterlin (2013) and Weeber, Kors & Mons (2005) have provided a detailed review of the different functions of mining tools available in the literature. Some researchers have successfully used text mining tools to identify candidate genes for diseases (Hristovski, Rindflesch & Peterlin, 2013; Karić & Karić, 2011).

In the present study, we integrate the text mining methodologies and other dataset (gene expression and drug target) to discover candidate genes or molecules that may play important roles in NHO caused by spinal cord and traumatic brain injury (SCI-TBI-HO). To the best of our knowledge, this is the first time this strategy has been applied in this area.

## Materials and Methods

### Discovery of potential SCI-TBI-HO mechanisms

**Figure 2:**
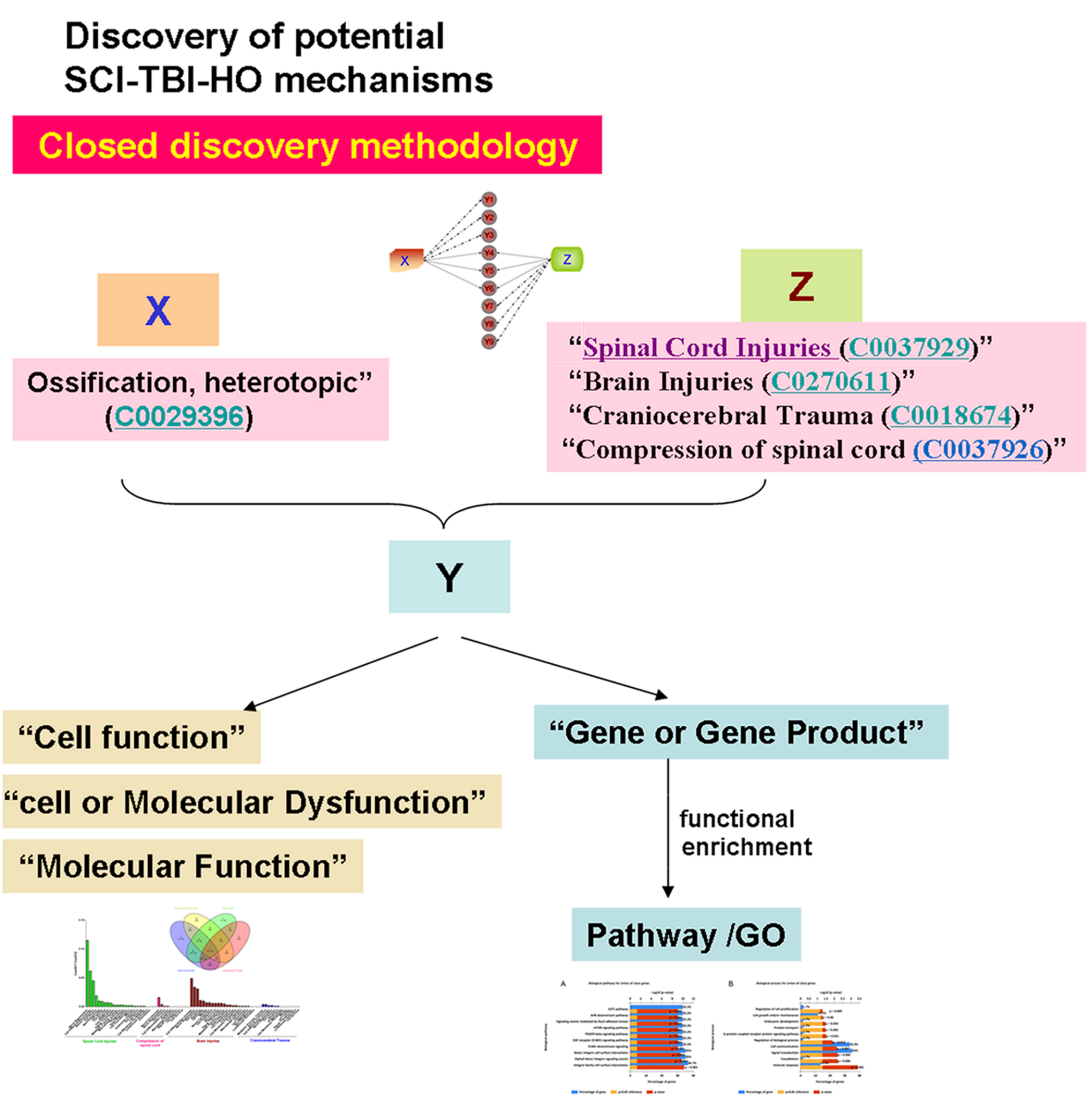
Discovery of potential SCI-TBI-HO mechanisms by closed discovery methodology. “Ossification, heterotopic” (C0029396) and “Spinal Cord Injuries (C0037929),” “Brain Injuries (C0270611),” “Craniocerebral Trauma (C0018674),” “Compression of spinal cord (C0037926)” were used as X and Z to mining the Y, including “Cell function,” cell or Molecular Dysfunction, Molecular Function, “Gene or Gene Product.” Enrichment analysis was adopted to detect the pathways and GO (Gene Ontology).

**Figure 3:**
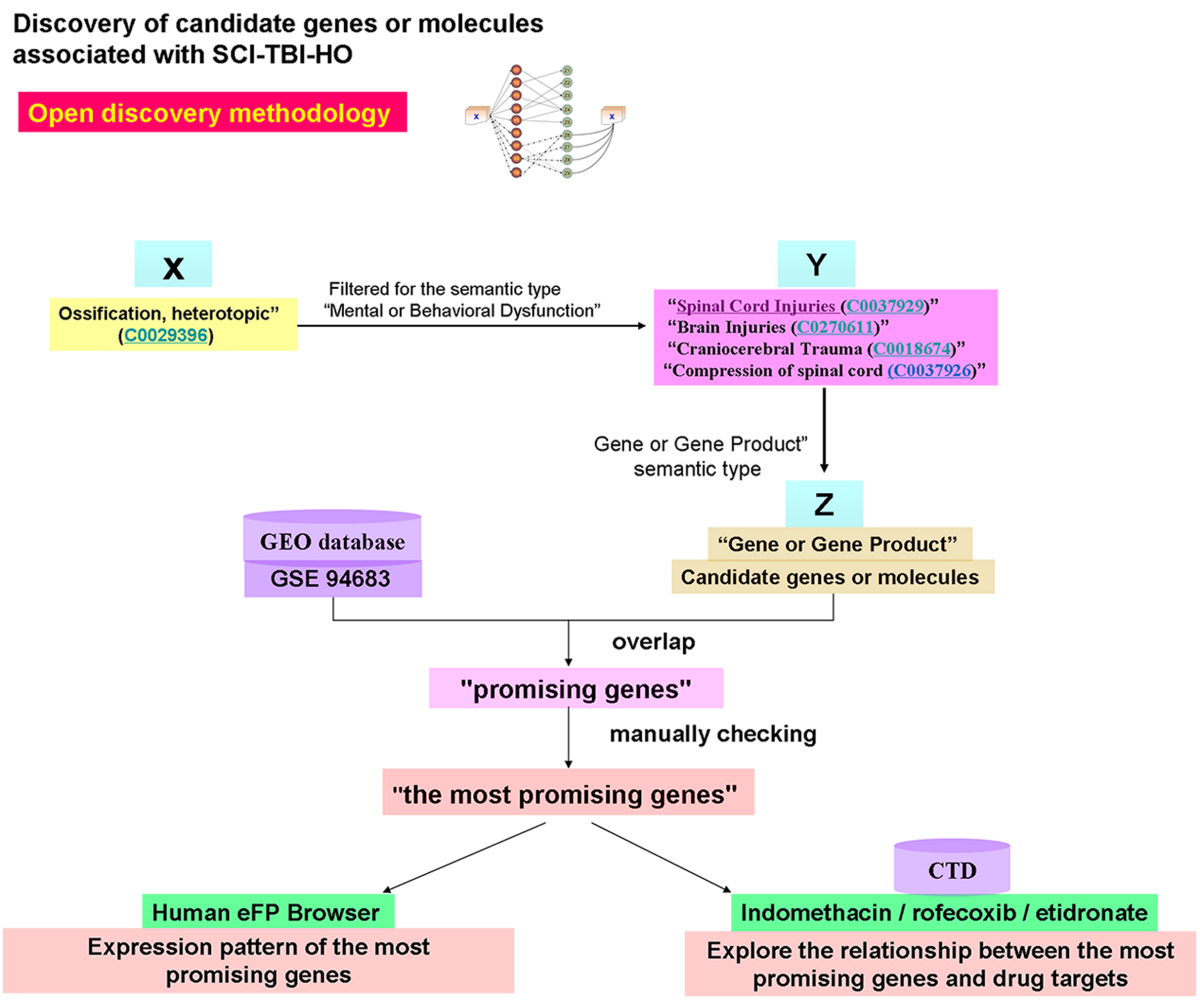
Discovery of candidate genes or molecules associated with SCI-TBI-HO by Open discovery methodology. “Ossification, heterotopic (C0029396)” (X) was used to screen the concepts of “spinal cord injury or traumatic brain injury” related to Heterotopic Ossification. Then the Concepts (Y) was used to mining the associated “Gene or gene product.” Second, the drug target was used to filter the “Z” by taking the intersection of them (define as promising genes). Finally, the Human eFP Browser was used to filter the promising genes.

BITOLA (Hristovski et al., 2006) (http://ibmi.mf.uni-lj.si/bitola) is an interactive literature-based biomedical discovery support system (Hristovski et al., 2006). This system features two types of methodology: closed and open discovery. The closed discovery methodology allows the input of two concepts (e.g., two disorders) and generates terms for potential explanations of their relationship (such as “Genes,” “Cell Function,” “Cell or Molecular Dysfunction,” and “Molecular Function”) (Hristovski et al., 2006). By contrast, the major role of the open discovery methodology is to mine new content (genes, drugs, or neuroscience, etc.) associated with the disease. Hence, we first deemed HO and spinal cord or traumatic brain injury as two concepts and attempted to explain the relationship between them by using the closed discovery methodology (Fig. 2). Secondly, we explored the candidate genes or molecules associated with NHO caused by spinal cord and traumatic brain injury (SCI-TBI-HO) using the open discovery methodology (Fig. 3).

The set of concepts available in the BITOLA system are currently Medical Subject Headings (MeSH), which are utilized to index Medline, and human genes from the Human Genome Organization (Hristovski et al., 2006). After searching for the keywords “Heterotopic ossification” and “Ectopic ossification,” we found only one concept, namely, “Ossification, heterotopic” (C0029396). We further verified this keyword by searching the MeSH database (https://www.ncbi.nlm.nih.gov/mesh/), and confirmed that the standard term for HO is “Ossification, heterotopic” (MeSH Unique ID: D009999). Therefore, “Ossification, heterotopic” was used for subsequent analysis.

Equivalent terms for the “Spinal cord and traumatic brain injury,” such as “Brain Injury (C0270611)” and “Compression of Spinal Cord,” indeed exist and are used in the literature. In order to incorporate other concepts that have a close relationship with, or are aliases for, spinal cord and traumatic brain injury, we searched for related disorders in BITOLA, as recommended. Subsequently, we included “Craniocerebral Trauma (C0018674)” and “Compression of Spinal Cord (C0037926),” which have a close relationship with spinal cord and traumatic brain injury, in the search (Table S1). Specifically, in this study, in order to enhance understanding of the molecular mechanisms for SCI-TBI-HO, we applied the “Semantic Type” filter for “Gene or Gene Product.” In order to find the cell and molecular functions for HO caused by spinal cord and traumatic brain injury, we applied the “Semantic Type” filter for “Cell Function” (defined as “mining molecular function”). Lastly, the intermediate “Y” concepts were retrieved.

It is generally recognized that pathway-based analysis may provide more meaningful information than gene-based analysis. Therefore, the relevant pathways for the “Gene or Gene Product” filtered terms were analyzed by functional enrichment using FunRich software (http://www.funrich.org) (Pathan et al., 2015). For ease of description, we define the “Brain Injuries (C0270611),” and “Craniocerebral Trauma (C0018674) as TBI-HO(caused by brain injury), and define “Spinal Cord Injuries (C0037929)” and “Compression of spinal cord (C0037926)” as SCI-HO (caused by spinal cord injury).

### Discovery of candidate genes or molecules associated with SCI-TBI-HO

As above, “Heterotopic ossification” was used as a starting concept (X), and “Spinal Cord Injuries (C0037929),” “Brain Injuries (C0270611),” “Craniocerebral Trauma (C0018674),” and “Compression of spinal cord (C0037926)” were used as the second concepts. Finally, the related “Z” terms (which here refer primarily to the “Gene or Gene Product” semantic type) were explored by defining the semantic type as “genes or molecules” and the semantic group as “Any.”

Via above steps, the “Gene or Gene Product” (“Z”) candidate genes/molecules were constructed, and this candidate genes/molecules set was used for the next analysis.

### Integration of gene expression profiles to filter the candidate genes or molecules

If the above candidate genes/molecules are expressed abnormally in the course of the NHO, it indicates that they may have important role in the progress of disease. Hence, we tentatively filtered and evaluated the “candidate genes/molecules” through the overview of gene expression levels (mRNA, Messenger Ribonucleic Acid) under different conditions (disease vs. control). Finally, we detected whether the candidate genes/molecules extracted by text mining matched with those different expression genes (DEGs). If they did, the candidate genes/molecules would be retained for next analysis, and if they did not, it would be excluded. We defined those genes that passed the filter criterion as “promising genes.”

#### Microarray data

Gene expression datasets were obtained from Gene Expression Omnibus (GEO) database. For the NHO, there was only one datasets (GSE94683) associated with the NHO. The more detail information about GSE94683 can refer to Torossian et al. (2017). Briefly, the platform used in GSE94683 was GPL10630 Agilent-021531 Whole Human Genome Oligo Microarray 4×44K (Feature Number version). The Whole Human Genome Oligo Microarrays were used to compare expression profiling of mesenchymal stromal cells (MSCs) from NHO patients and from bone marrow of healthy donors (control). Total RNA extracted from NHO-MSCs (7 samples) and Health-MSCs (9 samples) was used for cDNA array hybridization.

#### Statistical analysis of microarray data

Gene chip data of GSE94683 were analyzed by using BRB Array Tools (version 4.5.1 and R version 3.2.5; http://linus.nci.nih.gov/BRB-ArrayTools.html). The raw expression data were converted to log2 values and then normalized by using the quantiles normalization. In terms of spot filters, spots with intensity <10 were removed. The replicate spots within an array were averaged. Moreover, the genes under any of the following conditions were excluded: less than 20% of expression data have at least a 1.5-fold change in either direction from gene’s median value percent of data missing or filtered out exceeds 50%.

#### Analysis of differentially expressed genes

To identify the DEGs between NHO-MSCs and Health-MSCs, a random-variance *t*-test was used. Genes were considered highly significant if their *P* value was less than 0.001 and the false discovery rate (FDR) less than 0.05.

### Filtering of the intermediate concepts by manually checking

False-positive may existed due to the defects of literature mining itself. For example, the ambiguity of gene symbols (the symbol may refer to something other than the gene), is a known problem in text mining. Thus, we manually checked the “promising genes” to exclude the ambiguous term by reading the co-occurrence literature.

### Explore the relationship between the promising genes and drug targets

Clinically, multiple randomized controlled trials support the use of various NSAIDs for primary prevention of NHO following CNS injury (Banovac et al., 2004, 2001), indomethacin, rofecoxib, and etidronate being the most commonly used drugs. We identified the Anatomical Therapeutic Chemical (ATC) classes for indomethacin, rofecoxib, and etidronate using iATC-mHyb (Cheng et al., 2017) (http://www.jci-bioinfo.cn/iATC-mHyb). The target genes were extracted for these three drugs. Firstly, we used the Comparative Toxicogenomics Database (Davis et al., 2017) (CTD; http://ctdbase.org) to explore which genes/proteins interact with indomethacin, rofecoxib, and etidronate. Finally, we detected whether the “promising genes” were also matched with those target genes.

### Explore the expression pattern of the promising genes by tissue-specific gene data

The “promising genes” were further analyzed by tissue-specific gene data using the Human eFP Browser (Patel, Hamanishi & Provart, 2016). Clinically, the HO caused by traumatic brain injury always affects the joints of both upper and lower extremities, and HO caused by spinal cord injury always occurs below the level of the lesion. This ossification is also seen anteriorly, involving the iliopsoas and femoral neurovascular structures, laterally within gluteus minimus, and posteriorly extending from the ilium to the posterior femur encasing the sciatic nerve (Sullivan et al., 2013). The Human “electronic Fluorescent Pictograph” (eFP) Browser (Patel, Hamanishi & Provart, 2016) was used to interrogate whether the “promising genes” were most strongly expressed in these tissues.

## Results

### Discovery of potential SCI-TBI-HO mechanisms

Neurogenic heterotopic ossification is the ectopic formation of lamellar bone in non-osseous tissues following traumatic brain or medullar injury (Debaud et al., 2017). NHO is usually observed after insults such as spinal cord injury and traumatic brain injury (Genêt et al., 2011). Despite its known associations, etiology, and risk factors, the pathogenesis of HO remains poorly understood (Sakellariou et al., 2012).

Hence, we explored the potential mechanisms for SCI-TBI-HO using LBD.

In the closed discovery methodology, “Heterotopic ossification” was used as starting concept (similar to “A” in Swanson’s ABC model), and “Spinal Cord Injuries (C0037929),” “Compression of spinal cord (C0037926),” “Brain Injuries (C0270611),” and “Craniocerebral Trauma (C0018674)” used as end concepts (similar to “B” in Swanson’s ABC model).

**Figure 4:**
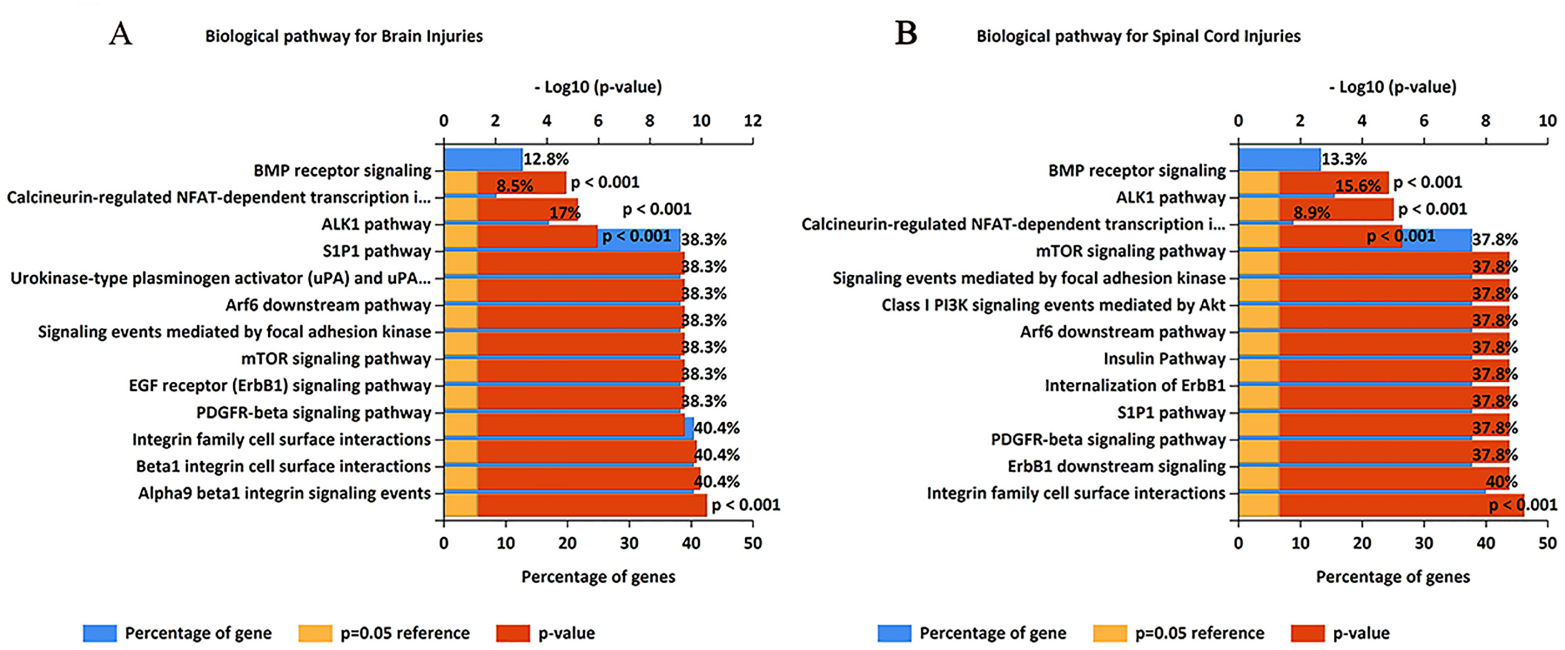
The enriched biological pathways for “Gene or Gene Product” terms identified using the closed discovery methodology. (A) Biological pathways enriched with TBI-HO-associated genes. (B) Biological processes enriched with SCI-HO-associated genes.

As results, 51 and 47 genes were “Gene or Gene Products” were suggested for TBI-HO and SCI-HO. respectively (Tables S2–S7) In total, 33 (50.8%) genes were overlapped between TBI-HO and SCI-HO. By functional enrichment analysis of these “Gene or Gene Product,” we found that 50 (89.3%) pathways were overlapped between TBI-HO and SCI-HO, including BMP receptor signaling, ALK1 pathway, Calcineurin-regulated NFAT-dependent transcription in lymphocytes, Integrin family cell surface interactions, Alpha9 beta1 integrin signaling events, Beta1 integrin cell surface interactions, Insulin Pathway, Internalization of ErbB1, Urokinase-type plasminogen activator (uPA) and uPAR-mediated signaling, PDGFR-beta signaling pathway, EGF receptor (ErbB1) signaling pathway, and Class I PI3K signaling events, and so on (Fig. 4A). However, some pathways appear only in one state, such as “ALK1 signaling events and Polyamines are oxidized to amines,” “aldehydes and H_2_O_2_ by PAOs” were enriched only for TBI-HO. Glucocorticoid receptor regulatory network and Glucocorticoid receptor signaling were enriched only for SCI-HO.

**Figure 5:**
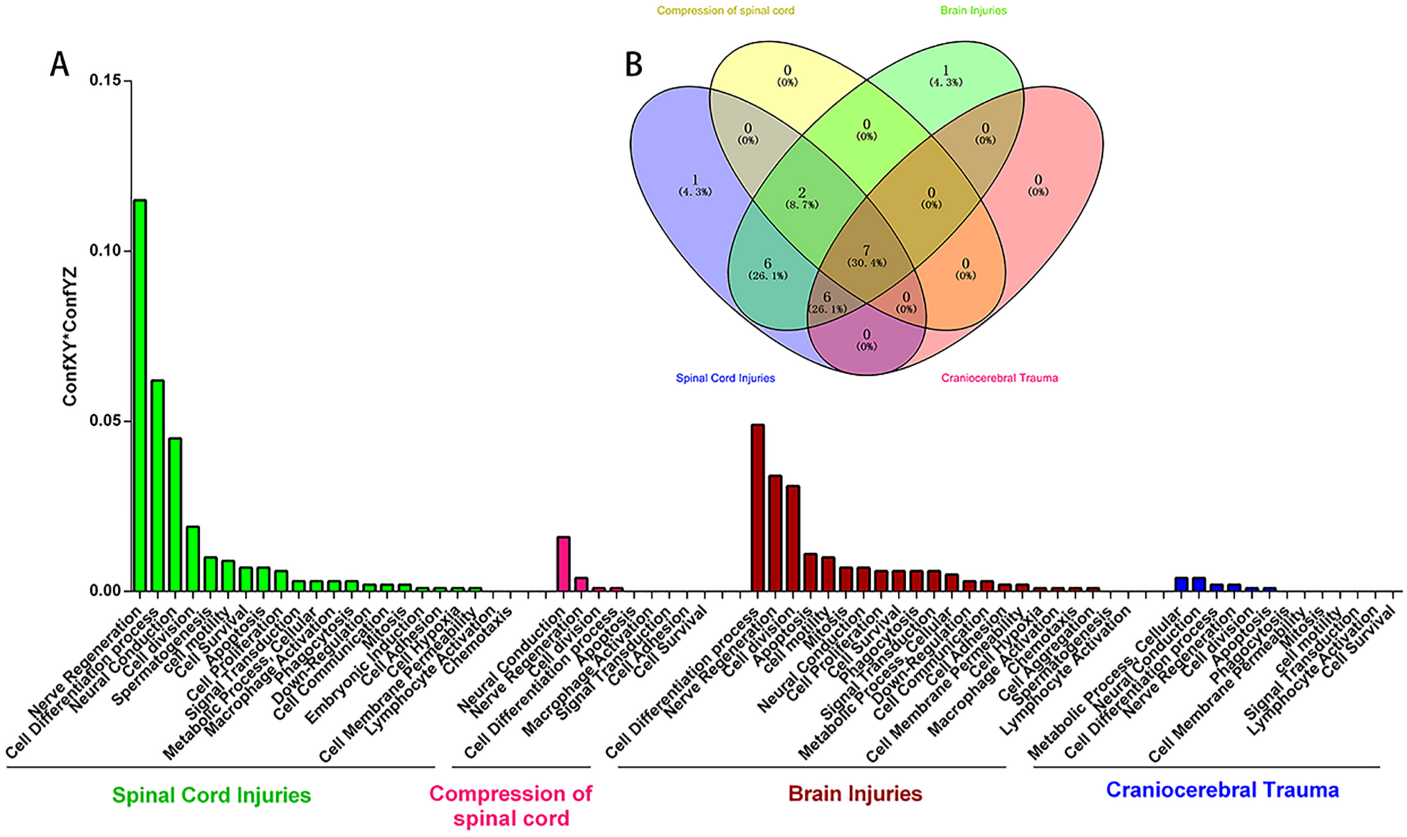
The extracted cell functions for “Spinal Cord Injuries,” “Compression of spinal cord,” “Brain Injuries,” and “Craniocerebral Trauma.” (A) Relative cell function for associations with “Spinal Cord Injuries,” “Compression of spinal cord,” “Brain Injuries” and “Craniocerebral Trauma.” (B) Overlap of the cell function among the four concepts.

In addition, we mined relative cell function for associations with SCI-TBI-HO. We showed that 22, 9, 22, and 13 terms related to cell function are associated with “Spinal Cord Injuries,” “Compression of spinal cord,” “Brain injuries,” and “Craniocerebral Trauma,” respectively (Tables S8–S10). Predictably, cell function was mainly involved in Nerve Regeneration, Cell Differentiation process, and Neural Conduction for Spinal Cord Injuries; Neural Conduction, Nerve Regeneration, and Cell division for Compression of spinal cord; Cell Differentiation process, Nerve Regeneration, Cell division, and Apoptosis for Brain Injuries; Metabolic Process, Cellular, Neural Conduction, Neural Conduction, Cell Differentiation process, and Nerve Regeneration for Craniocerebral Trauma (Tables S8–S10). An overlap of the four concepts, Spinal Cord Injuries,” “Compression of spinal cord,” “Brain injuries,” and “Craniocerebral Trauma,” yielded 7 terms: Nerve Regeneration, Cell Differentiation process, Neural Conduction, Cell division, Cell Survival, Apoptosis, and Signal Transduction (Fig. 5). However, the Embryonic induction were only for TBI-HO (caused by brain injury), and Cell Aggregation were only for SCI-HO (caused by spinal cord injury)

### Candidate genes or molecules derived from text mining

In the open discovery methodology, “Heterotopic ossification” was used as a starting concept, and “Spinal Cord Injuries (C0037929),” “Brain Injuries (C0270611),” “Craniocerebral Trauma (C0018674),” and “Compression of spinal cord (C0037926)” were defined as second concepts (similar to “B” in Swanson’s A to B to C model). In total, 526 “Gene or Gene Product” terms (candidate genes) for TBI-HO and 428 candidate genes for SCI-HO were found by the BITOLA system (Table S11).

### Differentially expressed genes

After data pre-processing and class comparison between different groups (NHO-MSCs vs. Health-MSCs), 187 DEGs were identified (Table S12). And 7 genes (*PTS*, *KCNN4*, *BAMBI*, *GDF15*, *LDLR*, *CCL2*, and *EBP*) were overlapped between the DEGs and the “Candidate genes or molecules” of TBI-HO, 3 genes (*CCL2*, *PTS* and *CLU*) were overlapped between the DEGs and the “Candidate genes or molecules” of SCI-HO.

### Manual checking

After manual checking, PTS were removed since the abbreviation or the alternative names of those genes used in the articles were not the original idea for the“gene or gene product.” PTS in the text is for “Posttraumatic seizures” (Zimmermann, Martin & Girgis, 2017) or “Physical therapists” (Gómara-Toldrà, Sliwinski & Dijkers, 2014) but not for the gene PTS (6-pyruvoyltetrahydropterin synthase) in the co-occurrence literature. Hence, PTS was removed and no further analysis. Therefore, the left 7 genes were seemed as promising genes.

### The promising genes were also the drug targets for indomethacin, rofecoxib, and etidronate

The consultation of the Comparative Toxicogenomics Database (Davis et al., 2017) revealed 2,028, 115, and 17 genes that interacted with indomethacin, rofecoxib, and etidronate, respectively (Tables S13–S15). In total, combining them, we obtained 2,115 target genes (Table S16). By comparing the 7 promising genes with the drug target genes, we found 4 genes (*GDF15, LDLR, CCL2*, and *CLU* seemed as most promising genes) that were present in both sets. Hence, our next step is to more deeply analyze the expression of these four genes in specific tissues

### Expression of the most promising genes in specific tissues

**Figure 6:**
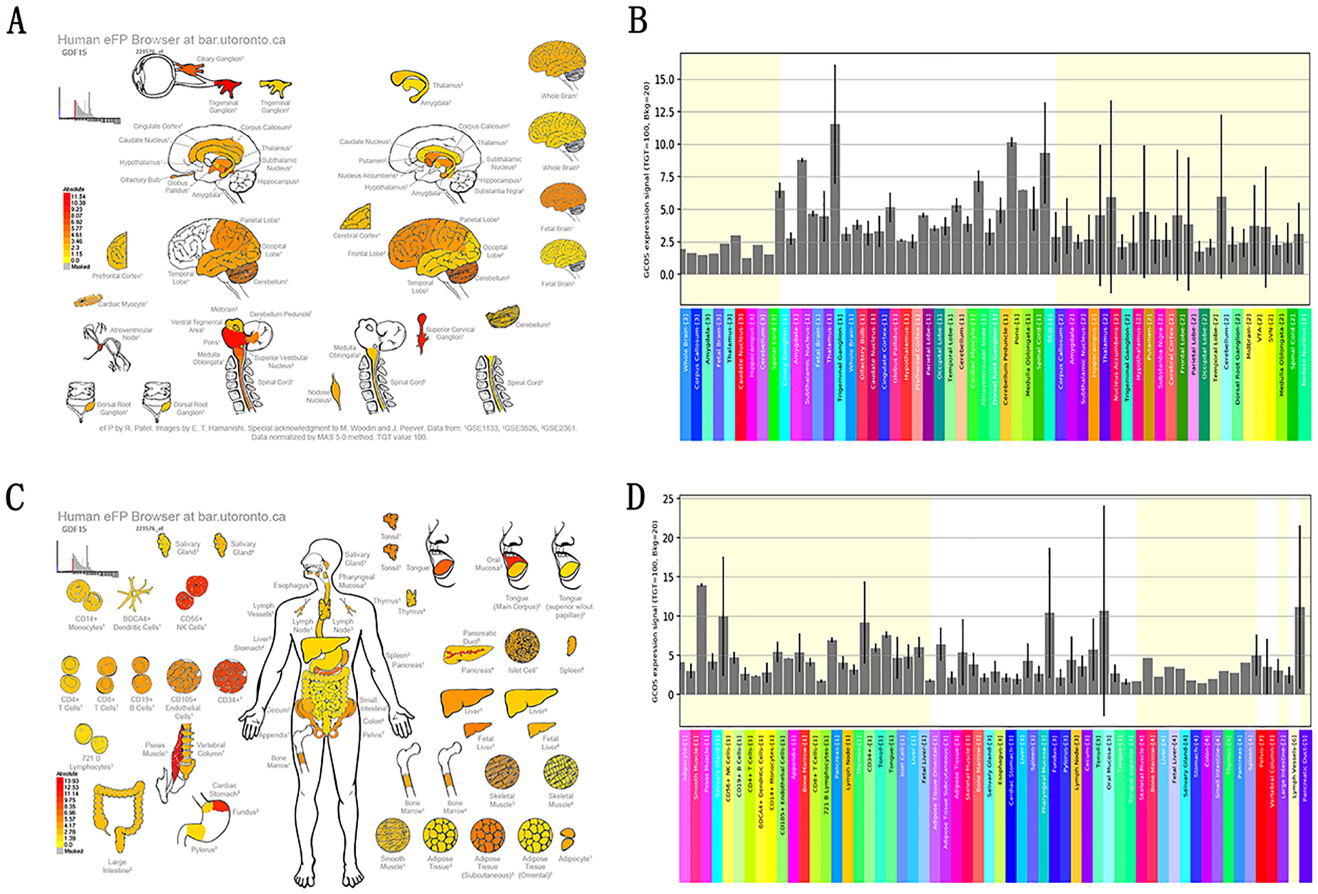
Expression and localization of the screened SCI-TBI-HO-associated genes. (A and C) Expression anatograms from the Human eFP Browser and (B and D) histograms of GDF15 expression in (A and B) “Nervous” tissues including brain and spinal cord and (C and D) the “Skeletal Immune Digestive” tissues, including smooth muscle, subcutaneous adipose tissue, adipose tissue, skeletal muscle, bone marrow, and lymphocytes (Patel, Hamanishi & Provart, 2016).

The analysis of tissue-specific gene expression may provide better explanations for each gene’s importance in the pathogenesis of HO caused by traumatic brain injury. We examined the gene expression patterns of the 4 most promising genes in specific tissues by using the Human eFP Browser (Patel, Hamanishi & Provart, 2016). These genes showed different expression patterns. In particular, we found that *GDF15, LDLR, CCL2* and *CLU* had higher expression in the brain and spinal cord, they were also expression in smooth muscle and skeletal muscle. The first example output shown in Fig. 6 is GDF15. Detailed information for other genes can be found in the Supplemental Files (Tables S17–S24).

## Discussion

NHO is a disorder of aberrant bone formation affecting one in five patients sustaining a spinal cord injury or traumatic brain injury (SCI-TBI-HO) (Sullivan et al., 2013). Once formed, the heterotopic bone typically needs to be excised surgically, which may result in wound healing complications, in addition to a risk of recurrence. Moreover, prevention remains challenging, and the cellular mechanisms of NHO remain unclear.

Disease gene identification is an important step in understanding the pathogenesis of the disease (Zhan et al., 2014). The expanding rate of knowledge of gene-disease associations can hardly keep pace with the rapidly growing body of biomedical literature. A considerable amount of useful biological or biomedical knowledge is buried in the form of free text, waiting to be discovered and transformed by researchers into more useful information (Liu, Liang & Wishart, 2015). Fortunately, text-mining technology and biomedical text extraction can largely overcome the issue of buried information.

In this study, text mining was used to generate potential explanations for the occurrence of SCI-TBI-HO. As results, 51 and 47 genes were “Gene or Gene Products” were suggested for TBI-HO and SCI-HO, respectively (Tables S2–S5). In total, 33 (50.8%) genes were overlapped between TBI-HO and SCI-HO. By functional enrichment analysis of these “Gene or Gene Product,” we found that 50 (89.3%) pathways were overlapped between TBI-HO and SCI-HO, including BMP receptor signaling, ALK1 pathway, Calcineurin-regulated NFAT-dependent transcription in lymphocytes, Integrin family cell surface interactions, Alpha9 beta1 integrin signaling events, Beta1 integrin cell surface interactions, Insulin Pathway, Internalization of ErbB1, uPA and uPAR-mediated signaling, PDGFR-beta signaling pathway, EGF receptor (ErbB1) signaling pathway, and Class I PI3K signaling events, and so on (Fig. 4A).

By using the closed discovery methodology and enrichment analysis, many pathways were found to be associated with TBI-HO and SCI-HO. In total, 50 (89.3%) pathways were overlapped between TBI-HO and SCI-HO. Among them, many pathways were related to integrin events, such as Integrin family cell surface interactions, Alpha9 beta1 integrin signaling events, and Beta1 integrin cell surface interactions. Generally, cells adhere to the substrate surface via integrins, a family of cell-surface receptors. Integrin-based attachment to the matrix plays a key role in physiological and pathological responses, such as development, tissue morphogenesis, adult tissue homeostasis, remodeling, and repair. Particularly, the disturbance of the extracellular matrix-integrin-cytoskeleton signaling axis often results in disease and tissue dysfunction (Docheva et al., 2014; Loeser, 2014). Moreover, normal adult articular chondrocytes express many subtypes of integrins, including α1β1, α3β1, α5β1, α10β1, αVβ1, αVβ3, and αVβ5 (Loeser, 2014); these integrins modify their expression pattern and regulate chondrocyte differentiation in the articular cartilage (Garciadiegocázares et al., 2015). Importantly, integrin signals can also play important roles as regulators of osteogenesis and mineralization (Shekaran et al., 2014; Di Maggio et al., 2017). On the other hand, joint and relative osteogenesis processes are always implicated in SCI-TBI-HO. Therefore, the integrin-based pathways suggested by our study may provide a further understanding of SCI-TBI-HO. Other pathways, such as the Insulin Pathway (Zhang et al., 2014), Internalization of ErbB1 (Zhang et al., 2011), uPA and uPAR-mediated signaling (Kalbasi Anaraki et al., 2015), PDGFR-beta signaling pathway (Tokunaga et al., 2008), EGF receptor (ErbB1) signaling pathway (Zhang et al., 2011), BMP receptor signaling and Class I PI3K signaling events (Kalbasi Anaraki et al., 2015), have been associated with osteogenesis and mineralization or HO. More interestingly, the PDGFR-beta signaling pathway has been implicated in familial idiopathic basal ganglia calcification, a brain disease characterized by anatomically localized calcifications in or near the blood microvessels (Betsholtz & Keller, 2014); hence, this pathway deserves further research, to comprehensively investigate its association with SCI-TBI-HO.

Reportedly, the pathophysiology of NHO in these two conditions is not the same (Yildiz & Ardiç, 2010, Reznik et al., 2014), after enrichment analysis, we found that some pathways appear only in one state, such as “Polyamines are oxidized to amines and aldehydes and H_2_O_2_ by PAOs” and “Interconversion of polyamines” were enriched only for TBI-HO. Polyamines have been implicated in a large number of cellular processes, including functioning of ion channels, nucleic acid packaging, DNA replication, apoptosis, transcription (Childs, Mehta & Gerner, 2003). More importantly, the polyamines, spermine and spermidine, both were significantly elevated during HO (Davis et al., 2016). We have also foud that “Glucocorticoid receptor regulatory network” and “Glucocorticoid receptor signaling” were enriched only for SCI-HO. It has been proved that glucocorticoid receptor expression was slightly increased at 15 min after spinal cord injury (Yan et al., 1999). However, glucocorticoid receptors expression was reported to decreased during 1–3 days after traumatic brain injury and increased 4–7 days (Hwang et al., 2006). Although not yet know whether there is different expression pattern of glucocorticoid receptors in HO caused by different reasons, those pathways provide new clues for detecting the different pathogenesis between TBI-HO and SCI-HO.

Previously, we have used a similar technique to that used in this study to detect the relative genes/candidate molecules in the interaction between oral lichen planus and depression (Zhan et al., 2017). We have innovatively used a text mining methodology alongside other dataset, including gene expression profiles data, drug target data and gene expression data of specific tissues, to explore promising genes for TBI-HO and SCI-HO.

We found three most promising genes (*GDF15*, *LDLR* and *CCL2*) for TBI-HO, 2 most promising genes (*CCL2* and *CLU*) for SCI-HO. These genes have been reported to play important roles in nerve injury, osteogenesis, or ossification. Among them, *GDF15* (growth differentiation factor 15), is noteworthy, mainly owing to its special expression pattern and function (Fig. 6). GDF15 is a distant member of the TGFβ superfamily and is described as a novel trophic factor for motor and sensory neurons (Ruschke et al., 2012). Reportedly, elevated GDF15 expression is a hallmark of many tissue injuries, GDF15 mRNA is upregulated in lesioned neurons and leasoned brain (Strelau et al., 2003; Schindowski et al., 2011). In the other hand, GDF15 can also induce BMSCs toward osteogenic lineage (Uchiyama et al., 2015). These findings imply that GDF15 may have a direct or indirect relationship in SCI-TBI-HO, which warrants further study. Low density lipoprotein receptor (LDLR) had been correlation with the calcification in neck vessels (Awan et al., 2011). The expression of LDLR was also changed during mouse brain development and after ischemic stroke (Rousselet et al., 2011). CCL2, C-C motif chemokine ligand 2, is one of several cytokine genes. The important role of CCL2 in the brain injury (mainly about traumatic brain injury) have been broadly reported (Semple et al., 2010; Liu et al., 2013; Luo et al., 2016). Moreover, CCL2 has been shown to be induced and involved in various neurodegenerative disorders including Alzheimer’s disease, multiple sclerosis, and ischemic brain injury (Semple, Kossmann & Morganti-Kossmann, 2010). Previously, macrophage contribution to HO has also been noted in vivo (Mosser & Edwards, 2008), macrophages are capable of secreting numerous pro-inflammatory cytokines and chemokines, including CCL2 (Convente et al., 2015). Combined with the topic of our study (NHO), it is reasonable to believe that this gene requires further study for NHO. CLU, also known as clusterin, is a complement inhibitor that appears to have a neuroprotective effect in response to tissue damage, and its expression is upregulated following acute head injury (Troakes et al., 2017). In addition, it has previously been observed that secreted clusterin protein inhibits osteoblast differentiation of bone marrow mesenchymal stem cells (Abdallah, Alzahrani & Kassem, 2018). CLU should also be given future focus in the study of SCI-TBI-HO.

## Conclusions

In conclusion, the underlying mechanisms of SCI-TBI-HO have been difficult to elucidate due to multiple factors. Using text mining, some pathways, such as the BMP receptor signaling, ALK1 pathway, Calcineurin-regulated NFAT-dependent transcription in lymphocytes, Integrin family cell surface interactions, Alpha9 beta1 integrin signaling events, Beta1 integrin cell surface interactions, Insulin Pathway, Internalization of ErbB1, uPA and uPAR-mediated signaling, PDGFR-beta signaling pathway, EGF receptor (ErbB1) signaling pathway, and Class I PI3K signaling events may serve as potential explanations for SCI-HO and TBI-HO, and enhance our understanding of its molecular mechanisms. The glucocorticoid receptors signal pathways provide new insights for detecting the different pathogenesis between TBI-HO and SCI-HO. Moreover, the four most promising genes, *GDF15*, *LDLR*, *CCL2* and *CLU* identified in this study, may offer new clues for researchers to plan targeted experiment for SCI-TBI-HO.

## Supplemental Information

10.7717/peerj.8276/supp-1 Supplemental Information 1 Genes or gene products were suggested by using the end concept "Spinal Cord Injuries," "Compression of spinal cord," "Brain Injuries" and "Craniocerebral Trauma." Table S1: Concepts of disorders related to Heterotopic Ossification (HO). Table S2: Genes or gene products were suggested by using the end concept "Spinal Cord Injuries." Table S3: Genes or gene products were suggested by using the end concept "Compression of spinal cord." Table S4: Genes or gene products were suggested by using the end concept "Brain Injuries." Table S5: Genes or gene products were suggested by using the end concept "Craniocerebral Trauma." Table S6: Union of genes or gene products among different end concepts. Table S7: Cell function related to Heterotopic ossification and "Spinal Cord Injuries."

10.7717/peerj.8276/supp-2 Supplemental Information 2 Genes or gene products were suggested by using the end concept "Spinal Cord Injuries," "Compression of spinal cord," "Brain Injuries" and "Craniocerebral Trauma." Table S8: Cell function related to Heterotopic ossification "Compression of spinal cord." Table S9: Cell function related to Heterotopic ossification "Brain Injuries." Table S10: Cell function related to Heterotopic ossification "Craniocerebral Trauma."

10.7717/peerj.8276/supp-3 Supplemental Information 3 The Intersection (close) of genes or gene products among different end concepts.

10.7717/peerj.8276/supp-4 Supplemental Information 4 Differentially expressed genes.

10.7717/peerj.8276/supp-5 Supplemental Information 5 The genes/proteins interact with Indomethacin, Rofecoxib and Disodium. Table S13: Genes/proteins interact with Indomethacin. Table S14: Genes/proteins interact with Rofecoxib. Table S15: Genes/proteins interact with Disodium etidronate. Table S16: Union of genes/proteins interact with Indomethacin, Rofecoxib, and Disodium etidronate.

10.7717/peerj.8276/supp-6 Supplemental Information 6 The expression of GDF15, LDLR, CCL2 and CLU in Nervous system. Table S17: The expression of GDF15 in Nervous system. Table S18: The expression of LDLR in Nervous system. Table S19: The expression of CCL2 in Nervous system. Table S20: The expression of CLU in Nervous system.

10.7717/peerj.8276/supp-7 Supplemental Information 7 The expression of GDF15, LDLR, CCL2 and CLU in Skeletal immune Digestive. Table S21: The expression of GDF15 in Skeletal immune Digestive. Table S22: The expression of LDLR in Skeletal immune Digestive. Table S23: The expression of CCL2 in Skeletal immune Digestive. Table S24: The expression of CLU in Skeletal immune Digestive.

## Additional Information and Declarations

### Data Availability

The following information was supplied regarding data availability: We text mined MEDLINE^®^, BITOLA System, GEO (for gene expression data), Comparative Toxicogenomics Database (drug target genes), and the Human eFP Browser (tissue-specific gene data). The complete text mining results are available in the Supplemental Tables.
